# 2022 Update of the Consensus on the Rational Use of Antithrombotics and Thrombolytics in Veterinary Critical Care (CURATIVE) Domain 1‐ Defining populations at risk

**DOI:** 10.1111/vec.13204

**Published:** 2022-05-02

**Authors:** Armelle deLaforcade, Lenore Bacek, Marie‐Claude Blais, Corrin Boyd, Benjamin M Brainard, Daniel L. Chan, Stefano Cortellini, Robert Goggs, Guillaume L Hoareau, Amy Koenigshof, Ron Li, Alex Lynch, Alan Ralph, Elizabeth Rozanski, Claire R Sharp

**Affiliations:** ^1^ Department of Clinical Sciences, Cummings School of Veterinary Medicine Tufts University North Grafton Massachusetts USA; ^2^ Bluepearl Specialty and Emergency Pet Hospital Tampa Florida USA; ^3^ School of Veterinary Medicine Murdoch University Murdoch Australia; ^4^ Department of Clinical Sciences University of Montreal Saint‐Hyacinthe QC Canada; ^5^ Department of Small Animal Medicine and Surgery, Clinical Sciences University of Georgia Athens Georgia USA; ^6^ Department of Clinical Science and Services The Royal Veterinary College London UK; ^7^ Department of Clinical Sciences Cornell University College of Veterinary Medicine Ithaca New York USA; ^8^ Department of Emergency Medicine University of Utah Salt Lake City Utah USA; ^9^ Department of Emergency Care Two by Two Animal Hospital Berrien Springs Michigan USA; ^10^ Department of Veterinary Surgical and Radiological Sciences School of Veterinary Medicine University of California Davis Davis California USA; ^11^ Department of Clinical Sciences NC State College of Veterinary Medicine Raleigh North Carolina USA; ^12^ MedVet‐New Orleans Metairie Louisiana USA

**Keywords:** arrhythmias, heartworm disease, protein‐losing enteropathy, vascular devices

## Abstract

**Objectives:**

To expand the number of conditions and interventions explored for their associations with thrombosis in the veterinary literature and to provide the basis for prescribing recommendations.

**Design:**

A population exposure comparison outcome format was used to represent patient, exposure, comparison, and outcome. Population Exposure Comparison Outcome questions were distributed to worksheet authors who performed comprehensive searches, summarized the evidence, and created guideline recommendations that were reviewed by domain chairs. The revised guidelines then underwent the Delphi survey process to reach consensus on the final guidelines. Diseases evaluated in this iteration included heartworm disease (dogs and cats), immune‐mediated hemolytic anemia (cats), protein‐losing nephropathy (cats), protein‐losing enteropathy (dogs and cats), sepsis (cats), hyperadrenocorticism (cats), liver disease (dogs), congenital portosystemic shunts (dogs and cats) and the following interventions: IV catheters (dogs and cats), arterial catheters (dogs and cats), vascular access ports (dogs and cats), extracorporeal circuits (dogs and cats) and transvenous pacemakers (dogs and cats).

**Results:**

Of the diseases evaluated in this iteration, a high risk for thrombosis was defined as heartworm disease or protein‐losing enteropathy. Low risk for thrombosis was defined as dogs with liver disease, cats with immune‐mediated hemolytic anemia, protein‐losing nephropathy, sepsis, or hyperadrenocorticism.

**Conclusions:**

Associations with thrombosis are outlined for various conditions and interventions and provide the basis for management recommendations. Numerous knowledge gaps were identified that represent opportunities for future studies.

**Abbreviations**
ADP: adenosine diphosphate
AF: atrial fibrillation
ATE: arterial thromboembolism
CURATIVE: Consensus on the Rational Use of Antithrombotics and Thrombolytics in Veterinary Critical Care
DIC: disseminated intravascular coagulation
HE: hepatic encephalopathy
IMHA: immune‐mediated hemolytic anemia
LOE: level of evidence
NRIMHA: nonregenerative immune‐mediated hemolytic anemia
PECO: Population Exposure Comparison Outcome
PLE: Protein‐losing enteropathy
PLN: protein‐losing nephropathy
PRCA: pure red blood cell aplasia
PSS: portosystemic shunt
PTE: pulmonary thromboembolism
PVT: portal vein thrombosis
TEG: thromboelastography
VAP: vascular access port

## 1 INTRODUCTION

Thrombosis continues to be recognized as a contributor to morbidity and mortality among companion animals experiencing a variety of disease processes, and there are a growing number of publications relating to the use of antithrombotics in veterinary medicine. The lack of veterinary studies quantifying risk factors for thrombosis in specific disease states complicates decision‐making regarding the use of antithrombotics. The 2019 Consensus on the Rational Use of Antithrombotics in Veterinary Critical Care (CURATIVE) guidelines examined populations at risk for thrombosis and defined a series of recommendations related to therapeutic use, monitoring, and discontinuation of antithrombotic therapy.^1^, ^2^, ^3^, ^4^, ^5^ A number of additional conditions potentially associated with thrombosis were considered in this second evaluation of the veterinary literature in an effort to promote best practices for the use of antithrombotics.

As in the previous iteration of the CURATIVE guidelines, the association between diseases and thrombosis was evaluated using a standard Population Exposure Comparison Outcome (PECO) question format. For conditions considered to be potentially associated with thrombosis, the veterinary literature was examined to determine whether in dogs or cats (Population, P), the development of a disease (Exposure, E), as opposed to remaining disease free (Comparison C), was associated with the development of thrombosis (Outcome, O). Recommendations for or against antithrombotic therapy based on risk for thrombosis were further refined using a Delphi survey process where CURATIVE group members were asked to agree or disagree with guideline recommendations and to suggest alternative wording as necessary. The results of the Delphi surveys and the resulting draft guidelines are available at supplementary data (S1). It should be noted that the CURATIVE Steering Committee made some minor wording changes to the draft guidelines for clarity and consistency after the Delphi surveys were completed, resulting in the final recommendations discussed below. Where appropriate, knowledge gaps were included to highlight specific areas of limitation and to encourage further investigation.

## 2 PECO QUESTION: HEARTWORM DISEASE (DOGS)

In dogs (P), is the development of heartworm disease (E), as opposed to remaining disease‐free (C), associated with the development of thrombosis (O)?

### 2.1 Guidelines

1.1 Heartworm (dogs)

a. Heartworm disease is associated with pulmonary artery thrombosis in dogs, with risk increasing with disease severity.
b. We recommend that antithrombotic therapy be considered in dogs with heartworm disease, particularly in thosewith more severe disease and those undergoing adulticide therapy.

### 2.2 Evidence summary

Most available evidence supported the PECO question (17 studies), with 2 studies considered neutral and none opposed. Proving thrombosis in clinical settings, particularly in the pulmonary arteries, is challenging, and studies were only included if a thrombus was confirmed by angiography, ultrasound, or histopathology. Studies reporting thrombin generation (ie, D‐dimers) and platelet reactivity were included to support or refute the association between dirofilariasis and a hypercoagulable state but with lower quality evidence scores. Overall, there is a strong association between clinical dirofilariasis and thrombosis and thromboembolism in dogs, with indications that this risk increases with disease severity, adulticide therapy and potentially with the presence of microfilaria.

Numerous studies document the presence of thrombi in dogs with dirofilariasis, universally in the same vascular bed as the worms. In a case series of 3 dogs with caval syndrome, 2 had extensive large and small vessel thrombosis throughout the pulmonary vasculature (LOE 5).^6^ In an experimental infection study of 20 healthy dogs (LOE 3), lung histopathology showed varying degrees of thrombi in all dogs, frequently associated with worm fragments.^7^ While most reports focus on the pulmonary vasculature, a small case series also documented worm‐associated thrombi in 3/5 dogs suffering from aberrant worm migration involving the aorta, medial iliac, and femoral arteries.^8^

Concentrations of D‐dimer are increased in up to 40% of heartworm‐infected dogs,^7^, ^9^ with higher concentrations seen in dogs with microfilaremia.^7^, ^10^ Similarly, plasma D‐dimer concentrations correlated with disease severity (LOE 3–5),^9^, ^11^, ^12^ increasing immediately after adulticide treatment (LOE 2),^11^ and decreasing with disease resolution (LOE 3).^12^, ^13^ Platelet reactivity is also increased in infected dogs (LOE 3),^14^, ^15^ with enhanced ^14^C‐serotonin release and platelet aggregation in response to collagen and adenosine diphosphate (ADP) in infected dogs. Infected dogs are also more likely to experience thrombocytopenia (< 150,000 platelets/μL) than noninfected dogs. Consistent with increased platelet reactivity, greater doses of aspirin, aspirin/dipyridamole or ticlopidine are needed to inhibit ADP‐induced aggregation in infected dogs, particularly following embolization of dead worms.^16^, ^17^ Necropsy lesions were less severe in dogs receiving ticlopidine than in those receiving no antiplatelet agent in one report (LOE 3).^17^ Antiplatelet therapy may diminish platelet adhesion, myointimal proliferation and vascular occlusion in infected dogs.^18^, ^19^

Adulticide treatment may increase thrombotic risk depending upon the protocol used, although older studies involving thiacetarsemide may be confounded by the thrombogenicity of the drug itself.^15^ Preceding adulticide treatment with doxycycline with or without ivermectin may lessen the number of arterial lesions compared to the administration of melarsamine without pretreatment (LOE 5).^20^ Treatment of dogs with patent infection following surgical worm implantation with a combination of imidacloprid, moxidectin, and doxycycline resulted in more thrombotic occlusion (on histopathology) than in untreated infected dogs (LOE 3).^21^ Thrombi were frequently associated with worm fragments, suggesting that this therapy resulted in worm death and subsequent embolism. In some dogs with preexisting dirofilariasis, pulmonary artery insertion of a large number (20‐50) of dead worms resulted in intimal proliferation of pulmonary arteries, dilation of main and lobar pulmonary arteries and obstruction of blood flow (LOE 3).^22^ The infusion of homogenized *Dirofilaria* antigen into the pulmonary arteries of dogs led to thrombosis within 1 hour of administration, but these thrombi were not detectable 5 days later, suggesting that Dirofilaria are thrombogenic but that actual worms are required for persistent thrombosis.^23^ The transient nature of these thrombi confirms the antigenicity of the heartworm but suggests that solid worm fragments are necessary to support prolonged thrombus residence.

### 2.3 Knowledge gap

Further investigation of the contribution of microfilariasis, especially to the thrombogenic risk of heart work disease, is warranted, particularly as the AHS does not currently recommend the use of antithrombotics in the management of dogs with heartworm disease.

## 3 PECO QUESTION: HEARTWORM DISEASE (CATS)

In cats (P), is the development of heartworm disease (E), as opposed to remaining disease‐free (C), associated with the development of thrombosis (O)?

### 3.1 Guidelines

1.2 Heartworm disease (cats)

a. Heartworm disease may be associated with pulmonary artery thrombosis in cats.
b. We suggest that antithrombotic therapy be considered in cats with heartworm disease, particularly in those with more severe disease or where other risk factors for thrombosis exist.

### 3.2 Evidence summary

Few studies of dirofilariasis in cats have evaluated thrombi or markers of ongoing thrombin generation. Two single case reports describe pulmonary arterial thrombi in cats with dirofilariasis, confirmed in one by histopathology.^24^, ^25^ A necropsy study of cats with naturally occurring dirofilariasis identified thrombi in 5/11 cats, although cats with thrombi often had comorbidities that might have contributed to thrombus formation (eg, lymphoma, hypertrophic cardiomyopathy, chronic nephritis).^26^ A single study of experimental heartworm infection described in 2 reports identified vascular occlusion and confirmed thrombi both with and without worm involvement.^27^, ^28^ One study, considered neutral to the PECO question, identified proliferation of the pulmonary artery tunica media in cats with dirofilariasis, resulting in vascular occlusion in the absence of identified thrombi.^29^ It is not clear if this represents a true absence of thrombi, or simply that they were not described. This report also suggests the possibility of increased vascular reactivity and remodeling in cats with heartworm disease that may clinically mimic thrombosis but without actual thrombi. Overall, the literature suggests that thrombosis is a potential complication of dirofilariasis in cats, but the prevalence of thrombosis is unclear, and the degree of risk is difficult to quantify. As such, we suggest that antithrombotic therapy be considered for cats with dirofilariasis, particularly in animals with severe disease or where other risk factors for thrombosis exist.

### 3.3 Knowledge gap

Studies with a primary aim of evaluating hemostatic changes and thrombotic complications in cats with dirofilariasis are needed to further characterize thrombogenic risk in this population, particularly as the American Heartworm Society does not currently recommend the use of antithrombotics in the management of cats with heartworm disease. In addition to characterizing the role of comorbidities, additional studies should differentiate the role of thrombosis versus vascular remodeling in causing vascular obstruction.

## 4 PECO QUESTION: IMMUNE‐MEDIATED HEMOLYTIC ANEMIA (IMHA) (CATS)

In cats (P), is the development of immune‐mediated hemolytic anemia (E), as opposed to remaining disease‐free (C), associated with the development of thrombosis (O)?

### 4.1 Guidelines

1.3 Immune‐mediated hemolytic anemia (IMHA) (cats)

a. Immune‐mediated hemolytic anemia in cats is weakly associated with pulmonary thromboembolism (venous thromboembolism).
b. There is no evidence that immune‐mediated hemolytic anemia is a risk factor for arterial thromboembolism in cats.
c. We suggest that antithrombotic therapy be considered in cats with immune‐mediated hemolytic anemia, where other risk factors for thrombosis exist.

### 4.2 Evidence summary

Only 1 study suggested an association between IMHA and pulmonary thromboembolism (PTE; venous thromboembolism) in cats. The point prevalence of IMHA in this study of feline PTE was 7% (2/29 cats).^30^ An association between IMHA and PTE was not substantiated by the other major retrospective study describing feline PTE.^31^ The 52 reports deemed neutral to the PECO question included 396 cats in total with suspected IMHA in all of its forms (primary and secondary IMHA, pure red blood cell aplasia [PRCA] and nonregenerative immune‐mediated hemolytic anemia [NRIMHA]). There was no report of thrombosis in any of these studies.^32^, ^33^, ^34^, ^35^, ^36^, ^37^, ^38^, ^39^, ^40^, ^41^, ^42^, ^43^, ^44^, ^45^, ^46^, ^47^, ^48^, ^49^, ^50^, ^51^, ^52^, ^53^, ^54^, ^55^, ^56^, ^57^, ^58^, ^59^, ^60^, ^61^, ^62^, ^63^, ^64^, ^65^, ^66^, ^67^, ^68^, ^69^, ^70^, ^71^, ^72^, ^73^, ^74^, ^75^, ^76^, ^77^, ^78^, ^79^, ^80^, ^81^, ^82^, ^83^ In addition, these 52 reports included multiple other studies describing arterial and venous thrombosis in cats. None of the cats in these studies had IMHA.^31^, ^45^, ^49^, ^54^, ^62^, ^66^, ^68^ Although 1 report suggested an association between IMHA and PTE in cats, the collective weight of the 52 neutral studies including nearly 400 cases suggests that there is either no association or a weak association between IMHA and thromboembolic complications in cats and that the overall risk of thrombosis (venous or arterial) in cats with IMHA appears low. No studies were identified that suggested evidence contrary to the PECO question. On the basis that IMHA might be weakly associated with venous thromboembolism, we suggest that antithrombotic therapy be considered in cats with IMHA, where other risk factors for thrombosis exist.

### 4.3 Knowledge gap

The mechanism explaining the differences between the thrombogenicity of IMHA in dogs versus cats is unknown.

## 5 PECO QUESTION: PROTEIN‐LOSING NEPHROPATHY (PLN) (CATS)

In cats (P), is the development of protein‐losing nephropathy (E), as opposed to remaining disease‐free (C), associated with the development of thrombosis (O)?

### 5.1 Guidelines

1.4 Protein‐losing nephropathy (PLN) (cats)

a. Protein‐losing nephropathy in cats is weakly associated with pulmonary thromboembolism (venous thromboembolism).
b. There is no evidence that protein‐losing nephropathy is a risk factor for arterial thromboembolism in cats.
c. We suggest that antithrombotic therapy be considered in cats with protein‐losing nephropathy, where other risk factors for thrombosis exist.

### 5.2 Evidence summary

Two studies suggest an association between PLN and PTE (venous thromboembolism) in cats.^30^, ^31^ The prevalence of PLN in these 2 reports of feline PTE was 6–14% (4/29 cats and 1/17 cats). By comparison, neoplasia (34‐35%) and cardiac disease (6‐41%) were more commonly associated with PTE in cats in the same reports. Several of the reports describing histopathologic changes in cats with PLN or glomerulonephritis had evidence of fibrin deposition; however, none of the studies that reported histopathology described any micro‐ or macrovascular thrombosis.^84^, ^85^ Numerous studies were considered neutral to the PECO question because they did not include a control group, they described arterial thrombosis and did not mention PLN, or they were reports focused on PLN that did not discuss or describe thrombosis.^45^, ^48^, ^49^, ^54^, ^59^, ^62^, ^63^, ^66^, ^67^, ^68^, ^70^, ^86^, ^87^, ^88^, ^89^, ^90^, ^91^, ^92^, ^93^, ^94^, ^95^, ^96^, ^97^, ^98^, ^99^, ^100^, ^101^, ^102^, ^103^, ^104^, ^105^, ^106^, ^107^, ^108^, ^109^, ^110^ Although these reports were considered neutral, their collective weight suggests that there is either no association or a very weak association between PLN and arterial thromboembolic complications in cats and that the overall risk of thrombosis in cats with PLN is seemingly low. No studies were identified that suggested evidence contrary to the PECO question. On the basis that PLN might be weakly associated with venous thromboembolism, we suggest that antithrombotic therapy be considered in cats with PLN, where other risk factors for thrombosis exist.

## 6 PECO QUESTION: LIVER DISEASE (DOGS)

In dogs (P), is the development of hepatic disease (E), as opposed to remaining disease‐free (C), associated with the development of thrombosis (O)?

### 6.1 Guidelines

1.5 Liver disease (dogs)

a. Liver disease is associated with thrombosis in a small subset of dogs only, independent of the specific underlying disease.
b. We suggest that antithrombotic therapy be considered in dogs with liver disease following an assessment of the risk and benefit in individual patients or where other risk factors for thrombosis exist.

### 6.2 Evidence summary

Of the 32 reports included for review, 13 suggest that liver disease may be associated with the development of thrombosis (1 LOE 5, good; 1 LOE 2, fair; 8 LOE 5, fair; 3 LOE 5, poor).

Hepatic disease might be preferentially associated with thrombosis of certain vessels, particularly the portal and splenic veins. Hepatic disease was documented in 29/140 (21%) dogs with portal or splenic vein thrombosis^111^, ^112^, ^113^, ^114^ but not in dogs with pulmonary or aortic thrombosis.^115^, ^116^, ^117^, ^118^, ^119^, ^120^ While this suggests an association between hepatic disease and thrombosis, comorbidities, including glucocorticoid administration, neoplasia, kidney disease or recent surgery, were common, and their contribution to overall thrombotic risk in these dogs could not be determined.^111^, ^112^, ^114^

Three studies employed thromboelastography (TEG) and documented a hypercoagulable state (but not thrombosis) in dogs with liver diseases, including extrahepatic biliary tract obstruction (10/10 dogs, 100%);^121^ chronic hepatopathies (7/21 dogs, 33,33%);^122^ and acute liver disease (2/21 dogs, 9,5%).^123^ These studies, while potentially relevant, do not directly address the PECO question.

Most studies were retrospective cohort studies or case series, typically without a control group. In one retrospective study, 8/96 (8%) dogs with liver disease that underwent CT angiography were diagnosed with portal vein thrombosis (PVT),^124^ while by comparison, 8/19 (42%) dogs with pancreatitis had PVT in the same study. This study also suggested that abdominal ultrasound may be insensitive for thrombosis detection compared to CT angiography, which suggests that studies using only abdominal ultrasound may underestimate the incidence of PVT.^125^ In a retrospective cohort study of 49 dogs with acute liver failure with or without signs of hepatic encephalopathy (HE), evidence of thrombosis was noted at necropsy in 4/23 (17.4%) dogs.^126^

In conclusion, because of the retrospective or in vitro nature of most of the studies, the impact of potential comorbidities on the association between liver disease and thrombosis is difficult to ascertain. Overall, the veterinary literature supports an association between liver disease and thrombosis, in particular PVT and splenic vein thrombosis, in a subset of dogs with liver diseases. We suggest that antithrombotic therapy should be considered in dogs with liver disease following an assessment of the risk and benefit in individual patients recognizing that bleeding disorders may be present due to severe liver dysfunction. The presence of other risk factors for thrombosis should also prompt clinicians to consider antithrombotics for dogs with liver disease.

### 6.3 Knowledge gap

Prospective controlled studies are needed to better ascertain the risk of thrombosis in dogs with liver disease of various etiologies and the contribution of comorbidities to the overall prothrombotic risk.

## 7 PECO QUESTION: LIVER DISEASE (CATS)

In cats (P), is the development of hepatic disease (E), as opposed to remaining disease free (C), associated with the development of thrombosis (O)?

### 7.1 Guidelines

1.6 Liver disease (cats)

No guidelines for this PECO question were generated during the current CURATIVE iteration. This important question will be addressed in a future iteration of the CURATIVE guidelines.

## 8 PECO QUESTION: CONGENTIAL PORTOSYSTEMIC SHUNTS (CPSS) (DOGS)

In dogs (P), is the presence of a congenital portosystemic shunt (E), as opposed to remaining disease‐free (C), associated with the development of thrombosis?

### 8.1 Guidelines

1.7 Portosystemic shunts (dogs)

a. Surgical correction of congenital portosystemic shunts (cPSS) in dogs may be associated with thrombosis in the postoperative period.
b. We suggest that antithrombotic therapy be considered in dogs undergoing surgical correction of cPSS, following an assessment of the risk and benefit in individual patients or where other risk factors for thrombosis exist.
c. We recommend against routine use of antithrombotic therapy in dogs with cPSS.

### 8.2 Evidence summary

Twelve reports met the criteria for review. Four reports supported the PECO question, with 7 considered neutral and 1 in opposition. In a retrospective study of 33 dogs with PVT (LOE 5, fair), 3 dogs had hepatic vascular anomalies, including 2 with cPSS,^111^ although the affected dogs had other risk factors, including recent splenectomy, the presence of an intravascular coil, portal hypertension or infectious disease. A retrospective case series of PVT (LOE 5, fair) reported thrombosis as a complication of cPSS ligation in 2 dogs.^127^ A case report of a dog with gallbladder infarction 48 hours after cPSS attenuation highlights the difficulty in determining the respective contribution to the risk of thrombosis of the cPSS and the surgical procedure.^128^

Various studies evaluated the hemostatic profiles of dogs with cPSS^.^ ^129^, ^130^, ^131^, ^132^ Most were deemed neutral to the PECO question because thrombosis was not investigated or reported. One study of dogs with cPSS (considered supportive of the PECO question) included TEG as a global coagulation assessment tool (LOE 5, good).^131^ This study showed that dogs with cPSS may be hypercoagulable despite clotting time prolongations, which supports hypocoagulability. Affected dogs also had decreased activities of the endogenous anticoagulants antithrombin and protein C. A prospective, observational study evaluating the diagnostic value of plasma protein C for detecting hepatobiliary disorders found that dogs with cPSS had significantly lower protein C activity than clinically ill dogs without cPSS (LOE 2, fair), potentially contributing to a hypercoagulable state.^133^ Thromboembolic disease was not reported in this study, although it was not the primary focus of the study and hence is considered neutral to the PECO question. Dogs with HE were more likely to be hypercoagulable on TEG and had higher fibrinogen concentrations. Dogs that developed PVT after cPSS ligation exhibited signs of HE prior to surgery.^127^ A study investigating the influence of cPSS on primary hemostasis found no clinically relevant alterations; hence, this study was judged to oppose the PECO question.^134^

Overall, there is some suggestion in the literature that cPSS increases the risk of thrombosis, particularly following surgical attenuation. These patients may be at risk of bleeding due to liver dysfunction; hence, antithrombotic therapy should be considered only after an assessment of the risk and benefit in individual patients, and routine administration of antithrombotic drugs for dogs with cPSS is not recommended.

## 9 PECO QUESTION: CONGENITAL PORTOSYSTEMIC SHUNTS (CPSS) (CATS)

In cats (P), is the presence of a congenital portosystemic shunt (E), as opposed to remaining disease free (C), associated with the development of thrombosis (O)?

### 9.1 Guidelines

1.8 Portosystemic shunts (cats)

a. Congenital portosystemic shunts (cPSS) may be associated with thrombosis in cats.
b. We recommend against routine use of antithrombotic therapy in cats with cPSS.
c. We suggest that antithrombotic therapy be considered in cats with cPSS, following an assessment of the risk and benefit in individual patients, when additional risk factors for thrombosis exist.

### 9.2 Evidence summary

Four reports met the criteria for review. Evidence from 1 (LOE 5, fair) documented a possible association of cPSS with PVT in cats.^62^ In this case series, 3/6 cats had congenital PSS at the time of PVT identification. One of these cats had recently undergone shunt ligation that may have increased thrombotic risk. In a multicenter retrospective study of 34 cats with cPSS, 11 cats developed complications, and 6 of these died. However, thrombosis was not described in any cat, and hence, this study was judged to oppose the PECO question.^135^ Two other studies (LOE 5, fair) identified hemostatic abnormalities in cats with cPSS, but neither described thrombosis.^136^, ^137^ These studies were considered neutral to the PECO question.

Overall, given the paucity of data to review, the risk of thrombosis associated with cPSS in cats cannot be clearly determined. As such, we recommend against routine use of antithrombotic therapy in cats with cPSS. Where more detailed assessments of risk versus benefit conducted in individual cats with cPSS are suggestive of thrombotic risk, where other risk factors for thrombosis are present, then we suggest antithrombotic therapy may be considered.

### 9.3 Knowledge gap

Studies are needed to investigate the potential association of thrombosis and PVT in cats prior to shunt ligation to remove the confounding effect of shunt ligation on the overall hemostatic state.

## 10 PECO QUESTION: CARDIAC ARRHYTHMIAS (DOGS)

In dogs (P), is the development of cardiac arrhythmias (E), as opposed to remaining disease‐free (C), associated with the development of thrombosis (O)?

### 10.1 Guidelines

1.9 Cardiac arrhythmias (dogs)

a. Atrial fibrillation may be associated with arterial thrombosis in dogs, particularly where reduced left atrial appendage flow velocity exists.
b. We suggest that antithrombotic therapy for atrial fibrillation in dogs should be considered, where other risk factors for thrombosis exist.
c. We recommend against the use of antithrombotic therapy in dogs with arrhythmias other than atrial fibrillation, unless other risk factors for thrombosis exist.

### 10.2 Evidence summary

Several reports of experimental models of rapid pacing‐induced atrial fibrillation (AF) in dogs were considered relevant to the PECO question. Two reports comparing the coagulability of blood sampled from the right atrium to that of peripheral blood showed evidence of hypercoagulability in atrial, but not peripheral, blood after induction of AF.^138^, ^139^ Another report demonstrated decreased trans‐mitral and left atrial appendage flow velocities during AF and following conversion to sinus rhythm compared to baseline measurements; this blood stasis may predispose to thrombosis.^140^ Induced AF in dogs also upregulates gene expression for some procoagulant mediators.^141^ One experimental study was classified as neutral to the PECO question, as it did not detect significant changes in von Willebrand factor or P‐selectin over time after the onset of induced AF, but the sample size was very small, and other coagulation parameters were not assessed.^142^ The only study deemed to oppose the PECO question showed no increase in the rate of thrombosis in 3 different canine models of AF following radiofrequency ablation compared to control dogs.^143^ While this opposes the PECO question, the relevance is likely impacted by the method of inducing AF.

Several potentially relevant case reports or case series were identified, but direct links between thrombosis and atrial fibrillation or other arrhythmias could not be made. One case report documented thrombosis in 3 dogs with AF,^144^ although 2/3 dogs had underlying mitral valve disease. In a case series of 39 Irish wolfhounds with congestive heart failure secondary to dilated cardiomyopathy and AF, 1 dog developed clinical signs consistent with arterial thromboembolism (ATE).^145^ A case series of 36 dogs with ATE documented AF in 1 dog, but ECG findings were not reported for all dogs.^118^ In a study of 7 dogs with thrombosis, 1 dog with biatrial thrombosis had ventricular tachycardia and acute myocardial failure of unknown origin listed as the diagnosis.^146^

Other case reports or case series were considered neutral to the PECO question because although thrombosis and arrhythmias were present concurrently, it was considered more likely that the arrhythmias developed subsequent to the thrombosis. In a case series of 16 dogs with splenic infarction, ventricular premature complexes were documented in 3 dogs,^114^ while in a report of 6 dogs with aortic or iliac arterial thrombosis, 1 dog was noted to have ventricular premature complexes.^147^ A single case report documented ATE and AF in a dog with hypothyroidism, but the case history implied that the ATE may have been present prior to atrial fibrillation.^148^

Overall, the available evidence suggests that AF may be associated with arterial thrombosis in dogs, particularly where reduced left atrial appendage flow velocity exists, or when electrical cardioversion is attempted and as such antithrombotic therapy should be considered for these dogs. For dogs with other arrhythmias, we do not recommend the use of antithrombotic therapy unless other risk factors for thrombosis exist.

### 10.3 Knowledge gap

The contribution of underlying structural cardiac changes to a prothrombotic state in dogs with cardiac arrhythmias remains unclear. Studies investigating hemostatic changes in dogs with cardiac arrhythmias in the absence of significant underlying cardiac disease and prior to the development of thrombosis are needed.

## 11 PECO QUESTION: CARDIAC ARRHYTHMIAS (CATS)

In cats (P), is the development of cardiac arrhythmias (E), as opposed to remaining disease free (C), associated with the development of thrombosis (O)?

### 11.1 Guidelines

1.10 Cardiac arrhythmias (cats)

a. Arrhythmias in cats with structural cardiac disease are associated with arterial thromboembolism.
b. We recommend the use of antithrombotic therapy for cats with arrhythmias and structural cardiac disease.

### 11.2 Evidence summary

Ten studies related to this PECO question were reviewed. Most reports identified in cats supported the PECO question, with only 1 neutral and none opposing. A small number of reports in cats directly investigated the link between thrombosis risk and AF or other arrhythmias. However, most cats in these reports had underlying cardiomyopathy, limiting the ability to draw conclusions about the thrombogenicity of arrhythmias in isolation. One case series reported data on 50 cats with AF.^149^ Aortic thromboembolism was documented at presentation in 7/50 cats, and a total of 8 cats died or were euthanized due to ATE; it is unclear whether the deaths include all 7 cats that had ATE on presentation. All cats in this study had underlying structural heart disease, predominantly cardiomyopathy with left atrial enlargement. An observational clinical study performed by Schober et al identified reduced left atrial appendage flow velocity, a known risk factor for thrombosis, in 9 cats with cardiomyopathy and AF.^150^ A single case of ATE and left atrial ball thrombus was reported in a cat with hypertrophic cardiomyopathy and AF.^151^ A case–control study was reviewed that identified arrhythmias as significant risk factors in univariate analysis for the outcome of composite cardiac death in cats with hypertrophic cardiomyopathy, where 34/107 cardiac deaths were due to ATE.^152^ However, arrhythmia did not remain in the authors’ multivariate model. A follow‐up to that study investigated the individual components of the composite outcome, and arrhythmia was not a significant predictor of death from ATE.^153^ However, the point estimate for the hazard ratio was above 1, and the confidence interval was wide, suggesting that further prospective studies are warranted to assess this relationship. Thus, this study was deemed neutral to the PECO question.

Most other relevant data in cats are contained in case series of cats with ATE or cardiomyopathy. These generally do not specifically investigate the association between arrhythmias and thrombosis. However, they demonstrate that the 3 conditions (ATE, cardiomyopathy, and arrhythmia) frequently coexist, so they were deemed to support the PECO question. In a case series of 127 cats with acute ATE, most of which had underlying cardiomyopathy, an arrhythmia was reported in 20 cats.^68^ In that series, 52 cats had ECG data reported, with 19 having arrhythmias, including ventricular premature complexes (13), AF (4), and atrial premature complexes (2). An older case series of 100 cats with ATE also detected abnormalities in 57 of the 67 cats that had an ECG performed, although this number included left atrial and ventricular enlargement patterns as well as true arrhythmias.^54^ In this study, common arrhythmias included isolated ventricular premature complexes (13), isolated superventricular premature complexes (13), AF (3), ventricular tachycardia (2), and supraventricular tachycardia (2). A case series of 41 cats with restrictive cardiomyopathy documented ATE in 17 cats.^154^ In that report, arrhythmias were recorded in 19 of the 34 cats that had an ECG performed, although it is not possible to determine whether arrhythmias were specifically related to ATE based on the data presented. Common arrhythmias in that study included atrial premature complexes (9), AF (5), ventricular premature complexes (5), and right bundle branch block (4). A case series of clinical and necropsy data from 12 cats with arrhythmogenic right ventricular cardiomyopathy documented postmortem mural thrombosis in 2 cats.^155^ Arrhythmias were identified in the 8 cats that had ECG performed, including ventricular premature complexes (6), right bundle branch block (5), AF (4), ventricular tachycardia (3), first‐degree AV block (2), and supraventricular tachycardia (1). It is not possible to determine from the report whether the cats with thrombosis had arrhythmias detected. An early case report of ATE in 5 cats identified an arrhythmia consistent with AF in one cat.^156^

In summary, arrhythmias in cats with structural cardiac disease are associated with arterial thromboembolism, although it is difficult to determine the isolated contribution of the arrhythmia to thrombotic risk in these cats. Given the strong association between structural cardiac disease, arrhythmias and thromboembolism in cats, we recommend antithrombotic therapy for all affected cats.

### 11.3 Knowledge gap

Further studies are needed to differentiate the contribution of arrhythmias to changes in coagulation status from those of underlying cardiomyopathy, cardiac dysfunction and blood flow alterations.

## 12 PECO QUESTION: SEPSIS (CATS)

In cats (P), is the development of sepsis (E), as opposed to remaining disease‐free (C), associated with the development of thrombosis (O)?

### 12.1 Guidelines

1.11 Sepsis (cats)

a. Sepsis is associated with the development of thrombosis in a small subset of cats.
b. We recommend against routine use of antithrombotic therapy in cats with sepsis.
c. We suggest that antithrombotic therapy be considered for cats with sepsis, following an assessment of the risk and benefit in individual patients or where other risk factors for thrombosis exist.

### 12.2 Evidence summary

Hemostatic abnormalities are commonly identified in cats with sepsis, but thrombosis is infrequently reported. Three retrospective studies including 160 cats with sepsis described just 2 cats (1.3%) with pulmonary thrombosis noted at necropsy.^157^, ^158^, ^159^ In aggregate from 2 studies of cats with PTE, 6.5% cats (3/46) had sepsis.^30^, ^31^ Sepsis was a common cause of disseminated intravascular coagulation (DIC) in cats, affecting 9/46 cats in 1 study.^160^ In 19/24 nonsurvivors necropsied in this study, intravascular fibrin deposition was identified, but the underlying conditions of cats that were examined postmortem were not listed, thereby limiting the quality of the evidence provided. A retrospective study of pulmonary histopathology in 148 cats with *Cytauxzoon felis* infection included 1 cat (0.7%) with pulmonary thrombi.^161^

Four studies were considered neutral to the PECO question. A study investigating hemostatic changes in cats with sepsis showed changes consistent with coagulation activation, such as reduced protein C activity and increased D‐dimers, but thrombosis was not described; hence, the study was deemed neutral to the PECO question.^162^ Another report described 10 cats undergoing adrenalectomy for hyperadrenocorticism. Two cats developed septic complications, and 1 experienced a fatal thromboembolic event. However, it is unclear whether thrombotic events occurred in cats with sepsis.^163^ Several case reports describe thrombotic complications of sepsis in cats, but multiple potential causes of thrombosis were present.^164^, ^165^

Overall, cats with sepsis commonly develop hemostatic abnormalities consistent with the activation of coagulation, but the incidence of thrombosis in cats with sepsis is low. In cats with sepsis that do develop thrombi, PTE is most described. The low overall incidence of thrombosis in cats with sepsis argues against routine use of antithrombotic therapy in these animals. We suggest that antithrombotic therapy be considered for cats with sepsis, particularly where other risk factors for thrombosis exist. Because cats with sepsis can develop DIC and experience clinical bleeding, antithrombotic medication should only be initiated after an assessment of the risk and benefit in individual patients.

### 12.3 Knowledge gap

Studies specifically investigating the development of clinically relevant thrombosis in cats (microvascular and macrovascular) are needed to better understand the risk factors for thrombotic complications in this patient population.

## 13 PECO QUESTION: PROTEIN‐LOSING ENTEROPATHY (PLE) (DOGS)

In dogs (P), is the development of protein‐losing enteropathy (E), as opposed to remaining disease free (C), associated with the development of thrombosis (O)?

### 13.1 Guidelines

1.12 Protein‐losing enteropathy (PLE) (dogs)

a. Protein‐losing enteropathy in dogs is moderately associated with thrombosis that can affect the venous or arterial system.
b. We recommend antithrombotic therapy for all dogs with protein‐losing enteropathy unless the risks (particularly gastrointestinal bleeding) are deemed to outweigh the potential benefit in individual patients.

### 13.2 Evidence summary

A total of 96 reports were reviewed for this PECO question. Of these, 27 provided evidence suggesting a moderate to strong link between a diagnosis of PLE in dogs and the development of thrombotic complications, many of them life‐threatening or fatal.^111^, ^112^, ^166^, ^167^, ^168^, ^169^, ^170^, ^171^, ^172^, ^173^, ^174^, ^175^, ^176^, ^177^, ^178^, ^179^, ^180^, ^181^, ^182^, ^183^, ^184^, ^185^, ^186^, ^187^, ^188^, ^189^, ^190^ The overall frequency of thrombosis reported in articles describing dogs with PLE was 46/1798 (2.6%), while the overall frequency of PLE in reports describing thrombosis was 9/699 (1.3%). This suggests that thrombosis is an important complication of PLE, even though PLE is not the most common cause of thrombosis seen in clinical practice. The retrospective nature (LOE 5) and the lack of a control group in these studies prohibits true risk assessment.

There were numerous reports that were considered neutral to the PECO question since they described canine PLE populations without mention of thrombotic complications or dogs with thrombosis without mention of PLE. ^49^, ^66^, ^113^, ^114^, ^115^, ^116^, ^118^, ^119^, ^120^, ^146^, ^147^, ^191^, ^192^, ^193^, ^194^, ^195^, ^196^, ^197^, ^198^, ^199^, ^200^, ^201^, ^202^, ^203^, ^204^, ^205^, ^206^, ^207^, ^208^, ^209^, ^210^, ^211^, ^212^, ^213^, ^214^, ^215^, ^216^, ^217^, ^218^, ^219^, ^220^, ^221^, ^222^, ^223^, ^224^, ^225^, ^226^, ^227^, ^228^, ^229^, ^230^, ^231^, ^232^, ^233^, ^234^, ^235^, ^236^, ^237^, ^238^, ^239^, ^240^, ^241^, ^242^, ^243^, ^244^, ^245^ Most reports of dogs with thrombosis identified an underlying disease other than PLE. Most of the articles describing dogs with PLE made no attempt to identify thrombosis formally, to explore the role of therapy with corticosteroids, or to study coagulation parameters. As therapy such as corticosteroids can represent an additional risk factor^1^ for thrombosis, the additive effects of multiple risk factors may impact overall coagulation status. Even some of the studies on PLE that identified thrombotic complications failed to provide details regarding the nature, extent, severity of the thrombus or any data regarding coagulation assessments performed. As such, the estimate of the incidence of thrombosis in PLE studies is most likely to be an underestimate of the risk. No studies were judged to be in opposition to the PECO question.

The overall weight of the studies available suggests a moderate to strong association between PLE and thrombosis. Both arterial and venous thrombotic complications were identified in dogs with PLE, although venous thrombosis was more common. As such, we recommend antithrombotic therapy for all dogs with protein‐losing enteropathy unless the risks (particularly gastrointestinal bleeding) are deemed to outweigh the potential benefit in individual patients.

### 13.3 Knowledge gap

The differences in coagulation status between dogs with PLE experiencing thrombosis and those that do not develop thrombosis are not clear, as most studies that describe thrombosis in dogs with PLE do not include hemostatic testing. The contribution of corticosteroid therapy to the overall prothrombotic risk is also not known.

## 14 PECO QUESTION: PROTEIN‐LOSING ENTEROPATHY (PLE) (CATS)

In cats (P), is the development of protein‐losing enteropathy (E), as opposed to remaining disease‐free (C), associated with the development of thrombosis (O)?

### 14.1 Guidelines

1.13 Protein‐losing enteropathy (PLE) (cats)

a. Protein‐losing enteropathy in cats is weakly associated with venous thromboembolism (pulmonary thromboembolism).
b. There is no evidence that protein‐losing enteropathy is a risk factor for arterial thromboembolism in cats.
c. We suggest that antithrombotic therapy be considered for cats with protein‐losing enteropathy, where other risk factors exist.

### 14.2 Evidence summary

A review of the literature identified 17 studies relevant to the PECO question. Only 1 retrospective study suggested an association between PLE and PTE (venous thromboembolism) in cats.^30^ The incidence of PLE in this study of PTE in cats was 14% (4/29). There were no control groups, however, and hence, the risk of thrombosis in cats with PLE cannot be properly assessed. An association between PLE and PTE was not substantiated by the other major retrospective study describing PTE in cats.^31^ The 15 reports deemed neutral to the PECO question included 4 retrospective studies (LOE 5) describing a total of 82 cats with PLE (encompassing inflammatory bowel disease, lymphangiectasia, and other causes) in which there was no report of thrombosis as a complication.^246^, ^247^, ^248^, ^249^ In addition, within these 15 articles were 9 reports describing a total of 838 cats experiencing arterial or venous thrombosis in cats, but none of the cats in these studies had PLE as an underlying disease.^31^, ^37^, ^45^, ^54^, ^62^, ^63^, ^66^, ^68^, ^162^ The final 2 reports that were neutral to the PECO question were retrospective studies of 57 cats receiving dalteparin and 231 cats with reduced blood antithrombin concentrations. These studies were of poor quality given that they were not designed to address the PECO question; however, none of these cats had PLE.^67^, ^70^ One study suggested that PLE may be associated with a risk of bleeding in cats based solely on coagulation testing and could be considered in opposition to the PECO question.^250^

Although 1 study did suggest an association between PLE and PTE in cats, the collective weight of the 15 neutral studies including 82 PLE cases suggests that there is a weak association between PLE and thromboembolic complications in cats and that the overall risk of thrombosis (venous or arterial) in cats with PLE is seemingly low. As such, we suggest that antithrombotic therapy be considered for cats with protein‐losing enteropathy, where other risk factors exist.

### 14.3 Knowledge gap

Prospective studies with control groups are needed to better ascertain any association between PLE and thrombosis in cats.

## 15 PECO QUESTION: HYPERADRENOCORTICISM (CATS)

In cats (P), is the development of hyperadrenocorticism (E), as opposed to remaining disease‐free (C), associated with the development of thrombosis (O)?

### 15.1 Guidelines

1.14 Hyperadrenocorticism (cats)

a. No evidence‐based recommendations can be made regarding the use of antithrombotic therapy in cats with hyperadrenocorticism.
b. We suggest that antithrombotic therapy should not be routinely used in cats with hyperadrenocorticism.

### 15.2 Evidence summary

The literature describing hyperadrenocorticism in cats is scarce, with most studies describing case series or case reports (LOE 5).^251^, ^252^, ^253^, ^254^, ^255^, ^256^, ^257^, ^258^, ^259^, ^260^, ^261^, ^262^, ^263^, ^264^, ^265^, ^266^ Some reports included necropsy results or the findings of CT or abdominal ultrasound imaging, but none were focused on identifying thrombosis. Across all studies reviewed, a total of 75 cats were reported to have hyperadrenocorticism, and only 1 cat was diagnosed with PTE, as described in a retrospective study of 7 cats with hyperadrenocorticism.^260^ The scant literature suggests that the incidence of hyperadrenocorticism in cats is seemingly low but precludes evidence‐based recommendations. As such, we suggest that antithrombotic therapy should not be routinely used in cats with hyperadrenocorticism.

## 16 PECO QUESTION: GLUCOCORTICOID ADMINISTRATION (CATS)

16

In cats (P), is glucocorticoid administration (E), as opposed to no glucocorticoid administration, (C), associated with the development of thrombosis (O)?

### 16.1 Guidelines

1.15 Glucocorticoid administration (cats)

a. No evidence‐based recommendations can be made regarding the use of antithrombotic therapy in cats receiving exogenous glucocorticoids.
b. We suggest that antithrombotic therapy should not be routinely used in cats receiving exogenous glucocorticoids.

### 16.2 Evidence summary

Few studies were relevant to the PECO question, and evidence for a causal relationship between exogenous corticosteroid administration in cats and thrombosis is scarce. In one retrospective observational study of 25 cats receiving exogenous corticosteroids, none developed evidence of thrombosis. ^263^ In a study of 6 cats with PVT, 2 (33%) were receiving oral prednisolone at the time of presentation. However, all cats had underlying hepatic disease precluding the establishment of a clear association between corticosteroid administration and thrombosis.^62^ Similarly, in another study (LOE 5, good), 2/44 cats with distal ATE had received corticosteroids in the weeks preceding the thrombotic event, but these cats also had comorbidities considered high risk for thrombosis.^267^ In a retrospective study of 29 cats with PTE, 8 (27.6%) had received recent corticosteroids.^30^

Given the lack of controlled studies in the current veterinary literature, no causal relationship can be determined between exogenous corticosteroids and an increased risk of thrombosis. However, based on the overall limited evidence supporting the PECO question with the corresponding high frequency of use of glucocorticoids, we suggest that antithrombotic therapy should not be routinely used in cats receiving this class of medication.

## 17 PECO QUESTION: INTRAVENOUS CATHETERS (DOGS)

In dogs (P), is the presence of an intravenous catheter (E), as opposed to no intravenous catheter (C), associated with the development of thrombosis (O)?

### 17.1 Guidelines

1.16 Intravenous catheters (dogs)

a. The risk of thrombosis associated with IV catheters in dogs is unknown.
b. We suggest that antithrombotic therapy should be considered in dogs with IV catheters only where other risk factors for thrombosis exist.

### 17.2 Evidence summary

A review of the literature yielded one report (LOE 2, fair quality) that was supportive of the PECO question.^268^ In that study, 50 dogs with cephalic peripheral catheters placed to enable repeated radiotherapy were monitored daily using vascular ultrasound. Eighteen dogs developed phlebitis, suggesting an incidence of 36%. Ten dogs with phlebitis developed evidence of local thrombosis (58%), suggesting an overall incidence of catheter‐related thrombosis of 20% (10/50). Vascular ultrasound of the cephalic vein was performed in each dog prior to and soon after IV catheter placement and then at regular intervals, allowing the enrolled dogs to serve as their own controls.

Most identified studies were experimental and designed to evaluate the degree of catheter‐related thrombus formation as opposed to systemic thrombosis; none were deemed good quality. Study designs varied, but most did not have a no‐catheter control group and worked from an assumption that intravenous catheters are thrombogenic in dogs. Most studies compared catheter materials or designs, protocols for implantation or flushing, or methods of detecting thrombosis, such as radiolabeling of platelets or fibrinogen. The outcomes assessed were typically catheter function or the appearance and weight of catheter‐associated thrombus postmortem. Assessments of the clinical relevance of thrombi are limited. Catheter‐associated thrombosis was generally described as substantial and common, but methods of assessing this were limited. ^269^, ^270^, ^271^, ^272^, ^273^, ^274^, ^275^, ^276^, ^277^, ^278^, ^279^, ^280^, ^281^, ^282^, ^283^, ^284^, ^285^, ^286^, ^287^, ^288^, ^289^ Two studies^271^, ^286^ documented evidence of PTE, but most were not designed to evaluate the presence of systemic thrombosis. The short duration of most experiments suggests that catheter‐associated thrombosis can occur rapidly in dogs.

Two experimental studies^290^, ^291^ deemed neutral to the PECO question evaluated catheter patency as the only assessment of potential thrombosis, with equivocal results. Two studies, judged to oppose the PECO question, evaluated surgically implanted silicon‐based central venous catheters. Both were conducted by the respective catheter manufacturers and were deemed at high risk of bias.^292^, ^293^ Differences between catheter materials and designs were frequently detected but were inconsistent between studies. All catheter materials have been associated with some degree of thrombosis, and the only catheter designs that featured minimal thrombosis were experimental and are not commercially available. Most studies also employed surgically placed central catheters, which limits the generalizability of their findings to clinical practice.

Four case series were judged LOE 5 and in support of the PECO question. Two studies focused on IV catheter complications, and thrombosis was reported in both.^294^, ^295^ A third described treatment of cases of fibrin sheaths formed on dialysis catheters,^99^ while another described cranial vena cava thrombosis and reported IV catheterization as a potential risk factor.^230^ Two case–control studies designed for other objectives also reported on cases with IV catheters and associated thrombosis.^296^, ^297^ These were deemed LOE 5 for relevance to this PECO question. Four case reports documented thrombosis associated with IV catheters^298^, ^299^, ^300^, ^301^ but were not controlled. One LOE 5 study that opposed the PECO question documented complications of long‐term silicone central venous catheters and did not report any instances of thrombosis. However, that study relied on a retrospective review of medical records and was thus judged poor quality.^302^

Overall, the literature suggests that IV catheters are likely thrombogenic in dogs, but the experimental nature of most studies and the lack of appropriate controls precludes determining the degree of risk. As such, it is difficult to make an evidence‐based recommendation for widespread antithrombotic use in dogs with IV catheters. Overall, we feel it is reasonable to consider IV catheterization as an additional risk factor for thrombosis in dogs that warrants consideration as an indication for antithrombotic drugs, particularly where other risk factors for thrombosis exist. Reassessments of the need for IV catheters should be performed regularly, and catheters should be removed as soon as they are no longer needed.

## 18 PECO QUESTION: INTRAVENOUS CATHETERS (CATS)

In cats (P), is the presence of an IV catheter (E), as opposed to no IV catheter (C), associated with the development of thrombosis (O)?

### 18.1 Guidelines

1.17 Intravenous catheters (cats)

a. The risk of thrombosis associated with IV catheters in cats is unknown.
b. We suggest that antithrombotic therapy should be considered in cats with IV catheters only where other risk factors for thrombosis exist.

### 18.2 Evidence summary

Most studies evaluated thrombosis as a cause of catheter occlusion rather than assessing systemic thrombotic complications of catheter placement and thus did not directly address the PECO question. Four experimental (LOE 3) studies were identified that were considered relevant to the PECO question. In a report of polyethylene catheters surgically placed into the caudal vena cava, thrombosis was observed in 13/14 cats within 2.5 hours of placement.^303^ This study was deemed fair quality but lacked a no‐catheter control group, and the catheter material is no longer used. A second study compared long‐term vascular access in 25 cats with surgically placed polyurethane jugular catheters with implanted vascular access ports (VAPs) in 42 cats featuring silicone catheters. Loss of catheter function was assumed to indicate thrombosis, which occurred in 12% of jugular catheters within 1 week.^304^ Another experimental study found no difference in coagulation test results between cats sampled via a jugular catheter and those sampled by jugular venipuncture,^305^ but this study did not investigate catheter‐associated thrombosis and was deemed neutral to the PECO question. A final experimental study found no evidence of thrombosis in 48 catheterization episodes in 6 cats and therefore opposed the PECO question. However, observation for thrombosis was confined to evaluations of catheter patency, and the relevance to the PECO question was limited.^306^

The remaining studies were case series or reports (LOE 5) that mostly supported the PECO question, although evidence quality and PECO question relevance were generally low. One case series documented complications of 100 polyurethane jugular central venous catheters in 12 cats and 81 dogs. Fourteen catheters failed to aspirate, suggesting possible catheter thrombosis, and 2 had evidence of venous thrombosis confirmed by palpation or ultrasound. This was the only clinical study to use standardized prospective data collection, but thrombosis was generally assumed from loss of catheter patency, and complications in cats were not separated from those in dogs.^294^ An older case series evaluated 300 polypropylene surgically placed jugular catheters in dogs and cats. Thrombus formation was noted on 5/300 catheters (1.7%), but it is unclear how this was determined, and again, dogs and cats were not differentiated.^295^ Polypropylene catheters are no longer used; hence, this study is of limited applicability to current clinical practice. Thrombosis of dialysis catheters was described in a case series of 8 cats, but the associated risk cannot be estimated because there was no information provided about the total population at risk. Thus, it is not clear if thrombosis is a common or rare complication of dialysis catheter placement.^99^ In a case series of 29 cats with necropsy‐confirmed PTE, IV catheterization was noted as a potential risk factor, with 21/29 cats having had IV catheters during the preceding hospitalization episode. However, the association between IV catheter placement and PTE is confounded by associations between the presence of an underlying disease (necessitating IV catheter placement) and thrombosis.^30^ A case report describes total parenteral nutrition extravasation from a polyurethane jugular central venous catheter after thrombosis of the jugular vein. However, it is not clear whether the thrombosis was associated with the catheter itself, parenteral nutrition infusion, or extravasation.^307^ A single clinical report was judged to oppose the PECO question, but this was a case series of 2 cats with long‐term silicone jugular catheters that was uncontrolled and deemed of poor quality.^302^

Overall, the available evidence indicates that thrombosis is a complication of IV catheterization in cats. However, the degree of risk for systemic complications of catheter‐related thrombosis cannot be estimated from the current literature. It is likely that catheter placement technique, material, location, and dwell‐time all affect the associated risk of thrombosis, but there is presently insufficient evidence to make evidence‐based recommendations for widespread antithrombotic use in cats with IV catheters. As with dogs, IV catheters can be considered to represent an additional risk factor for thrombosis in cats but one that warrants consideration as an indication for antithrombotic drugs only where other risk factors for thrombosis exist. Regular determinations of the ongoing need for all catheters should be part of clinical practice to reduce the risk of thrombosis by eliminating a potential risk factor.

## 19 PECO QUESTION: ARTERIAL CATHETERS (DOGS)

In dogs (P), is the presence of an arterial catheter (E), as opposed to no arterial catheter (C), associated with the development of thrombosis (O)?

### 19.1 Guidelines

1.18 Arterial catheters (dogs)

a. The risk of thrombosis associated with arterial catheterization in dogs appears to be low.
b. No evidence‐based recommendations can be made regarding the use of antithrombotic therapy in dogs with arterial catheters.
c. We suggest that antithrombotic therapy should not be routinely used in dogs with arterial catheters.

### 19.2 Evidence summary

Four studies (LOE 5, fair) were identified that evaluated the function of arterial catheters in dogs and reported subsequent complications such as thrombosis.^308^, ^309^, ^310^, ^311^ None of these reports included a relevant control population (dogs without arterial catheters); hence, all 4 studies were considered neutral to the PECO question. Additionally, the complication defined as ‘catheter occlusion’ was not characterized in all cases to distinguish potential causes such as intraluminal thrombosis, catheter kinking, arterial spasm or thromboembolism. This precludes accurate determination of the association between arterial catheter placement and thrombosis or quantification of the resultant risk.

In 2 studies, arterial catheters were in place only during anesthetic procedures,^310^, ^311^ while in the 2 remaining studies, some catheters remained in place postoperatively.^308^, ^309^ The association between catheter dwell‐time and complication rates was partially explored in 2 studies. The dwell time was median (min‐max) 23.8 h (4.5‐257) in dogs^308^ and median (min‐max) 7.7 h (0.9‐42.5) in dogs and cats.^309^ In dogs, no relationship between dwell time and complication rates was identified.

In a study of 267 arterial catheters placed in 213 dogs and 13 cats, 112 catheter sites were evaluated by an anesthesiologist after catheter removal.^309^ Most were removed in the ICU following anesthetic recovery (< 10 h dwell time). In 72/112, occurring at a median (min‐max) of 16.7 h (2.3‐124.3) after removal, no abnormalities were revealed. Although not explicitly stated, it is inferred that catheter sites were inspected for the presence of a pulse in 108 sites in dogs (dogs may have had more than 1 arterial catheter). No pulse was detected in 21 sites (inferred that all of these were in dogs). In 3 dogs, a knot was palpated on or under the skin, which likely (but not definitively) indicates thrombosis. No ischemic complications were noted in any patient.^309^

In a study of 198 arterial catheters in dogs, complications were noted in 38 (19%),^308^ although in 59 (30%), the reason for catheter removal was “no longer aspirates/flushes.” Loss of catheter function was not noted as a complication in this study; rather, complications were categorized as pain on flush/aspirate, swollen paw, cold paw and site reaction. In dogs, there were 21 instances of swollen paws and 3 instances of cold paws. Taken together, the instances of loss of function, swollen paw and cold paw might all indicate thrombosis, but this cannot be confirmed.^308^

Overall, the 4 identified studies were considered neutral to the PECO question due to a lack of contemporaneous unexposed controls with which to estimate risk, the heterogeneous nature of the clinical population and the lack of confirmation of thrombosis in many instances. Confounding of risk by the underlying disease process and the anesthetic and surgical procedures also impedes accurately attributing risk to the presence of the arterial catheter. As such, the current literature is insufficient to determine whether the use of arterial catheters predisposes dogs to thrombosis and hence precludes evidence‐based recommendations for antithrombotics. Overall, the risk of thrombosis in dogs with arterial catheters is seemingly low, and hence, we recommend against routine use of antithrombotic agents for dogs with arterial catheters.

## 20 PECO QUESTION: ARTERIAL CATHETERIZATION (CATS)

In cats (P), is the presence of an arterial catheter (E), as opposed to no arterial catheter (C), associated with the development of thrombosis (O)?

### 20.1 Guidelines

1.20 Arterial catheters (cats)

a. The risk of thrombosis associated with arterial catheterization in cats appears to be low.
b. No evidence‐based recommendations can be made regarding the use of antithrombotic therapy in cats with arterial catheters.
c. We suggest that antithrombotic therapy should not be routinely used in cats with arterial catheters.

### 20.2 Evidence summary

Only 3 studies (LOE 5) were identified that specifically evaluated arterial catheters in cats and aimed to evaluate complications such as thrombosis.^308^, ^309^, ^312^ None of these studies included a control group without an arterial catheter; hence, all 3 were considered neutral to the PECO question. As with dogs, the complication ‘catheter occlusion’ was not further characterized to determine or differentiate the underlying cause. This lack of confirmation of thrombosis precludes true determination of the risk of thrombosis posed by the use of arterial catheters. In most patients, arterial catheters were placed for monitoring arterial blood pressure during anesthesia with continued use postoperatively. The association between arterial catheters and dwell time was partially explored in these 3 studies. The dwell times were median (mix‐max) 12 h (3.5‐35);^308^ 3 h (1‐117);^312^ and 7.7 h (0.9‐42.5, dogs and cats combined).^309^ Complication rates were related to dwell time, but the exact nature of the complications was not characterized.

In a study of 13 cats, arterial catherization sites were checked following catheter removal in only 4 cats.^309^ In each case, a pulse was detected distally, indicating that the arteries remained patent, but the remaining catheter sites were not evaluated.

In a study that included 29 arterial catheters in cats, 2 catheters (7%) were removed due to a complication.^308^ Loss of catheter function where the catheter could not be flushed and failed to aspirate occurred 8 times (28%), while for 4 catheters (14%), a complication of “cold paw” was noted. Thrombosis is a possible cause for these various complications but was not confirmed.

The most likely occurrence of thrombosis associated with an arterial catheter in a cat was noted in the study by Mooshian et al. (LOE 5, fair), in which 1 cat suffered ischemic injury secondary to a coccygeal arterial catheter resulting in tail amputation.^312^ Histopathology was not conducted to confirm thrombosis; however, the underlying condition of this cat was not reported, which precludes assessment of the contribution of other risk factors.

One additional study identified arterial thrombosis in a cat associated with an infected arterial catheter.^313^ The infection was confirmed by bacterial culture, and thrombosis was confirmed by histopathology following amputation. However, in this case report, the relative contributions of the bacterial infection and the arterial catheter itself to the thrombosis cannot be determined. The case report demonstrates that arterial thrombosis can occur in association with arterial catheters in cats, but the comorbidity and the uncontrolled nature of the report means that this study must be considered neutral to the PECO question.

Overall, the 4 studies that were reviewed neither support nor oppose the PECO question. While a proportion of cats with arterial catheters may have developed thrombosis as a consequence of the catheter, the risk of thrombosis was not compared to a contemporaneous population of cats without arterial catheters, which precludes an assessment of relative risk. In cases where thrombosis did or may have occurred, it could not be discerned whether an underlying disease predisposed the cats to thrombosis or if the use of an arterial catheter augmented the risk. In conclusion, there are insufficient data to determine whether the use of arterial catheters predisposes cats to thrombosis; hence, no evidence‐based recommendations can be made. Overall, the risk of thrombosis in cats with arterial catheters is seemingly low, and hence, we recommend against routine use of antithrombotics for cats with arterial catheters.

## 21 PECO QUESTION: VASCULAR ACCESS PORTS (DOGS)

In dogs (P), is the presence of a vascular access port (E), as opposed to no vascular access port (C), associated with the development of thrombosis (O)?

### 21.1 Guidelines

1.20 Vascular access ports (dogs)

a. There is insufficient evidence to determine whether the use of vascular access ports in dogs increases the risk of thrombosis.
c. No evidence‐based recommendations can be made regarding the use of antithrombotic therapy in dogs with vascular access ports.

### 21.2 Evidence summary

Four studies (all LOE 5, poor) met the criteria for review. Given the small number and limited quality of the studies identified, there was insufficient evidence to support the PECO question. None of the studies had contemporaneous controls, and definitive causes of VAP failure were rarely identified. In instances where thrombosis was identified at the time of VAP removal, the potential that infection contributed to the thrombus could not be ruled out.^314^, ^315^ Moreover, most dogs had VAPs implanted to enable the management of neoplasia (either for the administration of chemotherapy ^316^ or sedation for radiation therapy,^314^, ^317^), which may increase the inherent risk for thrombosis. Additionally, VAPs were typically functional for months, suggesting that the risk of developing complications related to thrombosis is relatively low. Three studies (LOE 5, poor) were considered neutral to the PECO question,^314^, ^315^, ^317^ whereas 1 report might be interpreted as being opposed to the PECO question since none of the dogs had any evidence of thrombosis.^316^ No study reported coagulation testing, and the impact of variation in animal size, underlying conditions, catheter materials and sizes, and device flushing protocols was not considered. Overall, there is insufficient evidence to make recommendations on the use of antithrombotic therapy in dogs with VAP in the absence of other risk factors for thrombosis.

## 22 PECO QUESTION: VASCULAR ACCESS PORTS (CATS)

In cats (P), is the presence of a vascular access port (E), as opposed to no vascular access port (C), associated with the development of thrombosis (O)?

### 22.1 Guidelines

1.21 Vascular access ports (cats)

a. There is no evidence that the use of vascular access ports in cats is associated with an increased risk of thrombosis.
b. We suggest that antithrombotic therapy should not be routinely used in cats with vascular access ports.

### 22.2 Evidence summary

Six studies describing the use of VAPs in cats were reviewed, the largest of which included 46 cats (and 126 dogs) receiving radiotherapy for various forms of neoplasia.^317^ Permanent loss of catheter patency was among the complications described, but the mechanism and species were not characterized, limiting the relevance of these data to the PECO question. Another study noted thrombotic complications in 2/6 cats with femoral vein VAPs.^315^ In 1 cat, a clinically silent thrombus distal to the catheter tip was identified that did not limit device utility. In the second cat, thrombosis was related to catheter kinking that necessitated removal. Another study described surgically placed VAPs in 42 healthy cats, 2 of which developed fatal pulmonary thromboembolism.^304^ An additional 3 studies including a total of 36 cats with implanted VAPs reported no thrombotic complications.^318^, ^319^, ^320^ The lower quality clinical studies (LOE 5, poor) that lacked a control group^315^, ^317^ were considered neutral to the PECO question because the risk of thrombosis from VAP implantation was frequently confounded by underlying neoplasia. In the experimental studies (LOE 3 good‐poor),^318^, ^319^, ^320^ involving healthy cats, no risk of thrombosis was apparent, and hence, these studies were assessed as opposing the PECO question. Only 1 study (LOE 3, fair) suggested an association between VAP implantation and thrombosis in cats,^304^ but another group of cats experienced no thrombotic complications, thereby limiting the strength of the association. Overall, we suggest that antithrombotic therapy not be routinely used in cats with VAP.

## 23 PECO QUESTION: EXTRACORPOREAL CIRCUITS (DOGS)

In dogs (P), is the use of an extracorporeal circuit (E), as opposed to no extracorporeal circuit (C), associated with the development of thrombosis (O)?

### 23.1 Guidelines

1.22 Extracorporeal circuits (dogs)

a. Extracorporeal circuits are associated with activation of coagulation and circuit thrombosis in dogs, necessitating use of systemic or regional anticoagulation during extracorporeal procedures unless otherwise contraindicated.
b. The risk of systemic thrombosis in dogs between extracorporeal therapy cycles appears low.
c. We suggest that antithrombotic therapy should not be routinely used between extracorporeal therapy cycles in dogs unless indicated by other risk factors for thrombosis.

### 23.2 Evidence summary

Extracorporeal circuits, specifically those used for hemodialysis and therapeutic apheresis, are associated with activation of platelets, neutrophil‐platelet aggregation, and activation of coagulation. Extracorporeal circulation leads to reduced and turbulent blood flow within the circuit, high shear stress, and blood contact with both air and artificial surfaces (eg, filter membranes, tubing, IV catheter lumens). Platelet activation in response to shear stress leads to platelet‐neutrophil aggregate formation,^321^ and a study utilizing radiolabeled canine platelets circulated through a dialyzer showed that platelets exposed to the circuit were significantly more thrombogenic than healthy control platelets.^322^ Leukocyte activation triggered by contact between blood and extracorporeal circuits contributes to a procoagulant state.^323^ In some instances, contact activation by the artificial membrane may also occur.^323^ Given these circuit‐induced procoagulant effects, anticoagulation (systemic or regional) during extracorporeal therapy is required to avoid clotting within the circuit. We therefore recommend that anticoagulation (systemic or regional) be used during the procedure unless otherwise contraindicated.

Veterinary studies describing extracorporeal techniques consistently describe thrombotic complications related to the filter or the central venous catheter. However, across all studies reviewed, no systemic thrombotic complications were noted in dogs undergoing membrane therapeutic plasma exchange (n = 51),^324^, ^325^, ^326^ intermittent hemodialysis (n = 183),^327^, ^328^ or centrifugal apheresis (n = 4).^329^ Anticoagulation strategies, when used, were limited to the duration of the session and varied among the studies reviewed. The overall quality of evidence was judged to be low because of the retrospective nature of the studies and the lack of monitoring for systemic thrombosis. Several studies of cardiopulmonary bypass in dogs involving extracorporeal circulation reported thrombotic complications. However, all dogs in these reports had confounding factors for thrombosis, including underlying diseases, prosthetic vascular implants and cardiac surgical procedures.

Overall, despite the known procoagulant impact of the extracorporeal circuit, the risk of systemic thrombosis in dogs undergoing extracorporeal therapy appears to be low within and between cycles. We therefore suggest that antithrombotic therapy should not be routinely used between extracorporeal therapy cycles in dogs unless indicated by other risk factors for thrombosis. However, it should be recognized that no study in dogs directly addresses the PECO question because of the presence of confounding factors, including underlying disease and central venous catheters.

## 24 PECO QUESTION: EXTRACORPOREAL CIRCUITS (CATS)

In cats (P), is the use of an extracorporeal circuit (E), as opposed to no extracorporeal circuit (C), associated with the development of thrombosis (O)?

### 24.1 Guidelines

1.23 Extracorporeal circuits (cats)

a. Extracorporeal circuits are associated with activation of coagulation and circuit thrombosis in cats, necessitating the use of systemic or regional anticoagulation during extracorporeal procedures unless otherwise contraindicated.
b. No evidence‐based recommendations can be made regarding the use of antithrombotic therapy between extracorporeal therapy cycles in cats.

### 24.2 Evidence summary

Two studies investigating dialysis catheter performance in cats were reviewed.^99^, ^330^ One report assessed patency of a nitric oxide releasing extracorporeal circuit using an in vivo feline model,^330^ and the second evaluated changes in blood flow rate through a dialysis catheter in response to tissue plasminogen activator in 17 dogs and 8 cats.^99^ Neither study was designed to investigate systemic thrombosis; hence, neither directly addressed the PECO question. Although the use of extracorporeal circuits in cats necessitates regional or systemic anticoagulation as it does in dogs, there is insufficient evidence upon which to base recommendations for the use of antithrombotics in cats between extracorporeal therapy cycles.

### 24.3 Knowledge gaps

No studies were identified that directly addressed the PECO question, despite ample evidence supporting the high incidence of thrombosis in people undergoing extracorporeal circulation as opposed to those who do not.

## 25 PECO QUESTION: TRANSVENOUS PACEMAKER (DOGS)

In dogs (P), is the presence of a transvenous pacemaker (E), as opposed to no transvenous pacemaker (C), associated with the development of thrombosis (O)?

### 25.1 Guidelines

1.24 Transvenous pacemaker (dog)

a. The presence of a transvenous cardiac pacemaker is weakly associated with symptomatic thrombosis in dogs, while lead‐associated thrombosis is more common.
b. We recommend the use of antithrombotic therapy in dogs following transvenous pacemaker implantation, where other risk factors for thrombosis exist.
c. We suggest that antithrombotic therapy be considered in all dogs following transvenous pacemaker implantation.

### 25.2 Evidence summary

Thirty‐five references that included ≥1 dog with transvenous pacemaker implantation were reviewed.^244^ ^.^ ^331^, ^332^, ^333^, ^334^, ^335^, ^336^, ^337^, ^338^, ^339^, ^340^, ^341^, ^342^, ^343^, ^344^, ^345^, ^346^, ^347^, ^348^, ^349^, ^350^, ^351^, ^352^, ^353^, ^354^, ^355^, ^356^, ^357^, ^358^, ^359^, ^360^, ^361^, ^362^, ^363^, ^364^ Most were case series or single case reports (LOE 5), and 1240 dogs were described, of which 60 had suspected or confirmed thrombosis (5%). Two studies were experimental (LOE 3) but of poor quality.^336^, ^361^ In most dogs, the thrombi caused no clinical signs, and the thrombi were identified at necropsy or noted using echocardiography. Only 17 dogs had clinically significant thrombi described (∼1%).

Overall, 13 studies (1 LOE 3, 12 LOE 5) support the PECO question and suggest an association between transvenous pacemakers and thrombosis in dogs.^244^, ^331^, ^335^, ^338^, ^341^, ^344^, ^345^, ^355^, ^357^, ^358^, ^360^, ^361^, ^362^ The one LOE 3 report that supported the PECO question was a retrospective case series of 101 dogs that had polyurethane insulated transvenous pacemaker leads implanted.^361^ These pacemakers were in place for between 10 days and 13 years before euthanasia and postmortem examination. Thrombi were detected in 34/101 dogs, but no dogs showed clinical signs. Nine studies (LOE 5, fair) supported the PECO question, including 3 case series focused on pacemaker complications.^335^, ^341^, ^355^ In total, these three studies described thrombi in 7 dogs out of 426 dogs with sufficient follow‐up to analyze complication rates. Thrombi occurred in the cranial vena cava, pulmonary arterial tree and aorta. It is unclear whether any of the dogs with thrombosis had additional predisposing comorbidities. The remaining supportive studies included 17 dogs with thrombosis following transvenous pacemaker implantation, typically in the cranial vena cava, some of which were fatal. Some studies reported comorbidities, including trauma, PLN and infection, that could have contributed to the risk of thrombosis.^244^, ^331^, ^338^, ^344^, ^345^, ^357^, ^358^, ^360^, ^362^, ^363^ Four publications (LOE 5) were considered neutral to the PECO question either due to lack of follow‐up, multiple possible causes for thrombosis, or reasons other than the presence of a pacemaker that were deemed likely to result in thrombosis (e.g., stenosis).^334^, ^337^, ^339^, ^346^

Eighteen studies (1 LOE 3 good, 17 LOE 5 good‐fair) were judged to be neutral to the PECO question based on long‐term follow‐up of dogs after pacemaker implantation without reports of thrombotic complications.^332^, ^333^, ^336^, ^340^, ^342^, ^343^, ^347^, ^348^, ^349^, ^350^, ^351^, ^352^, ^353^, ^354^, ^356^, ^359^, ^363^, ^364^ An experimental (LOE 3) study described 74 dogs with implanted pacemakers (≤180 days). A variety of complications were reported, but thrombosis did not occur. ^336^ The other studies were focused on pacemaker complications and included large sample sizes (total n = 380), and thrombosis was not reported^.328, 350–352^ One case series of dogs in which pacemakers were implanted for the management of suspected myocarditis described 74 dogs with prothrombotic comorbidities, including protein‐losing disease, immune‐mediated disease, the presence of spontaneous echocontrast, hepatic disease, and hyperadrenocorticism. Thrombosis was not reported as a complication, with a median survival time of 1079 days.^359^

Overall, the available literature suggests that the presence of a transvenous pacemaker in dogs is associated with the development of thrombosis, with an estimated overall prevalence of ∼5% and an estimated prevalence of symptomatic thrombosis of ∼1%. Cranial vena cava syndrome is the most commonly associated thrombotic complication of transvenous pacemakers in dogs, with outcomes ranging from resolution to death. The risk of thrombosis is not uniform, however, because some dogs with other comorbidities that might predispose to thrombosis did not develop thrombotic complications from their pacemaker. It seems reasonable, however, that all dogs with pacemakers and prothrombotic comorbidities should receive antithrombotic therapies for prophylaxis, while antithrombotic therapy can be considered in every dog following transvenous pacemaker implantation to minimize the risk of symptomatic thrombosis development.

### 25.3 Knowledge gap

Additional studies are needed to better characterize the role of comorbidities in the development of thrombosis in dogs with transvenous pacemakers.

## 26 PECO QUESTION: TRANSVENOUS PACEMAKER (CATS)

In cats (P), is the presence of a transvenous pacemaker (E), as opposed to no transvenous pacemaker (C), associated with the development of thrombosis (O)?

### 26.1 Guidelines

1.25 Transvenous pacemaker (cats)

a. No evidence‐based recommendations can be made regarding the use of antithrombotic therapy in cats following transvenous cardiac pacemaker placement.

### 26.2 Evidence summary

Most studies of cardiac pacemaker implantation in cats describe epicardial lead placement only (total n = 52) and were not further evaluated. Five studies consisting of isolated case reports or small case series (LOE 5, fair) describing cats with implanted transvenous pacemakers were reviewed,^365^, ^366^, ^367^, ^368^, ^369^ although 2 studies included cats with epicardial leads.^366^, ^367^ Four studies were considered neutral to the worksheet question since thrombosis was not described, while 1 was considered neutral because chylothorax developed as a complication, and thrombosis could not be ruled out as the cause. ^369^ The case report that was considered neutral to the PECO question described a cat with a three‐week history of syncope due to third‐degree atrioventricular block.^369^ A permanent transvenous pacemaker was placed in the left jugular vein, and the cat was asymptomatic for 3 months following implantation, when chylothorax was detected that required repeated thoracocentesis and ultimately prompted euthanasia. The cause of chylothorax was not determined, but thrombosis was a potential explanation. Overall, since most literature describes epicardial lead placement and there is no definitive evidence of thrombosis in cats following transvenous pacemaker placement, we were unable to make evidence‐based recommendations on the use of antithrombotic medications for these cats.

## Supporting information

Supplementary data (S1). Delphi survey results for Domain 1 PECO questions: Defininig populations at risk
