# Supplementary material for: 2022 Update of the Consensus on the Rational Use of Antithrombotics and Thrombolytics in Veterinary Critical Care (CURATIVE) Domain 1‐ Defining populations at risk
Source: J Vet Emerg Crit Care (San Antonio). 2022 May 2;32(3):289–314. doi: 10.1111/vec.13204 (PMC9322658; doi:10.1111/vec.13204)
Supplement: Supplementary file 1 — Supplementary data (S1). Delphi survey results for Domain 1 PECO questions: Defininig populations at risk [file VEC-32-289-s001.docx]

1.1. Heartworm (Dogs) [16/16 Round 2]

a. Heartworm disease is associated with pulmonary artery thrombosis in dogs, with risk increasing with disease severity.

b. We recommend that antithrombotic therapy be considered in dogs with heartworm disease, particularly in those with more severe disease and those undergoing adulticide therapy.

1.2. Heartworm (Cats) [16/16 Round 2]

a. Heartworm disease may be associated with pulmonary artery thrombosis in cats.

b. We suggest that antithrombotic therapy can be considered in cats with heartworm disease, particularly in those with more severe disease or where other risk factors for thrombosis exist.

1.3. Immune-mediated hemolytic anemia (IMHA) (Cats) [19/19 Round 1]

a. Immune-mediated hemolytic anemia in cats is weakly associated with pulmonary thromboembolism (venous thromboembolism).

b. There is no evidence that immune-mediated hemolytic anemia is a risk factor for arterial thromboembolism in cats.

c. We suggest that antithrombotic therapy can be considered in cats with immune-mediated hemolytic anemia, particularly where other risk factors for thrombosis exist.

1.4. Protein-losing nephropathy (PLN) (Cats) [19/19 Round 1]

a. Protein-losing nephropathy in cats is weakly associated with pulmonary thromboembolism (venous thromboembolism).

b. There is no evidence that protein-losing nephropathy is a risk factor for arterial thromboembolism in cats.

c. We suggest that antithrombotic therapy can be considered in cats with protein-losing nephropathy, particularly where other risk factors for thrombosis exist.

1.5. Liver disease (Dogs) [16/16 Round 2]
a. Liver disease is associated with thrombosis in a small subset of dogs only, independent of the specific underlying diagnosis.
b. We suggest that antithrombotic therapy can be considered in dogs with liver disease following an assessment of the risk and benefit in individual patients, or where other risk factors for thrombosis exist.

1.6 Liver disease (Cats) No worksheet

1.7. Portosystemic shunt (PSS) (Dogs) [16/16 Round 2]

a. Surgical correction of congenital portosystemic shunts in dogs may be associated with thrombosis in the postoperative period.

b. We suggest that antithrombotic therapy can be considered in dogs undergoing surgical correction of portosystemic shunt, following an assessment of the risk and benefit in individual patients, or where other risk factors for thrombosis exist.

c. We recommend against routine use of antithrombotic therapy in dogs with portosystemic shunt.

1.8. Portosystemic shunt (PSS) (Cats) [16/16 Round 2]

a. Congenital portosystemic shunts may be associated with thrombosis in cats.

b. We suggest that antithrombotic therapy can be considered in cats with congenital portosystemic shunts, following an assessment of the risk and benefit in individual patients, or where other risk factors for thrombosis exist.

c. We recommend against routine use of antithrombotic therapy in cats with portosystemic shunt.

1.9. Cardiac arrhythmias (Dogs) [15/16 Round 2, 1 disagreed due to lack of evidence]
a. Atrial fibrillation may be associated with arterial thrombosis in dogs, particularly where reduced left atrial appendage flow velocity exists, or when electrical cardioversion is attempted.
b. We suggest that antithrombotic therapy for atrial fibrillation in dogs should be considered, especially when electrical cardioversion is attempted, or where other risk factors for thrombosis exist.

c. We recommend against the use of antithrombotic therapy in dogs with arrhythmias other than atrial fibrillation, unless other risk factors for thrombosis exist.

1.10. Cardiac arrhythmias (Cats) [15/16 Round 2, 1 felt it was unclear that arrhythmia increased risk above presence of structural cardiac disease]

a. Arrhythmias in cats with structural cardiac disease are associated with arterial thromboembolism.

b. We recommend the use of antithrombotic therapy for cats with arrhythmias and structural cardiac disease.

1.11. Sepsis (Cats) [16/16 Round 2]
a. Sepsis is associated with the development of thrombosis in a small subset of cats.

b. We recommend against routine use of antithrombotic therapy in cats with sepsis.

c. We suggest that antithrombotic therapy can be considered for cats with sepsis, following an assessment of the risk and benefit in individual patients, or where other risk factors for thrombosis exist.

1.12 Protein-losing enteropathy (PLE) (Cats) [19/19 Round 1]

a. Protein-losing enteropathy in cats is weakly associated with venous thromboembolism (pulmonary thromboembolism).

b. There is no evidence that protein-losing enteropathy is a risk factor for arterial thromboembolism in cats.

c. We suggest antithrombotic therapy can be considered for cats with protein-losing enteropathy, particularly where other risk factors exist.

1.13 Protein-losing enteropathy (PLE) (Dogs) [19/19 Round 1]

a. Protein-losing enteropathy in dogs is moderately associated with thrombosis that can affect the venous or the arterial system.

b. We recommend antithrombotic therapy for all dogs with protein-losing enteropathy unless the risks (particularly of gastrointestinal bleeding) are deemed to outweigh the potential benefit in individual patients.

1.14. Hyperadrenocorticism (Cats) [16/16 Round 2]

a. No evidence-based recommendations can be made regarding the use of antithrombotic therapy in cats with hyperadrenocorticism.

b. We suggest antithrombotic therapy should not be routinely used in cats with hyperadrenocorticism.

1.15. Glucocorticoid administration (Cats) [16/16 Round 2]

a. No evidence-based recommendations can be made regarding the use of antithrombotic therapy in cats receiving exogenous glucocorticoids.

b. We suggest that antithrombotic therapy should not be routinely used in cats receiving exogenous glucocorticoids.

1.16. Intravenous catheters (Dogs) [16/16 Round 2]

a. The risk of thrombosis associated with intravenous catheter placement or presence in dogs is unknown.

b. Reassessments of need for intravenous catheters should be performed at least daily, and catheters removed as soon as they are no longer needed.

c. We suggest that antithrombotic therapy should be considered in dogs with intravenous catheters only where other risk factors for thrombosis exist.

1.17. Intravenous catheters (Cats) [16/16 Round 2]

a. The risk of thrombosis associated with intravenous catheter placement or presence in cats is unknown.

b. Reassessments of need for intravenous catheters should be performed at least daily, and catheters removed as soon as they are no longer needed.

c. We suggest that antithrombotic therapy should be considered in cats with intravenous catheters only where other risk factors for thrombosis exist.

1.18. Arterial catheters (Dogs) [16/16 Round 2]

a. The risk of thrombosis associated with arterial catheterization in dogs appears to be low.

b. No evidence-based recommendations can be made regarding the use of antithrombotic therapy in dogs with arterial catheters.

c. We suggest antithrombotic therapy should not be routinely used in dogs with arterial catheters.

1.19. Arterial catheters (Cats) [16/16 Round 2]

a. The risk of thrombosis associated with arterial catheterization in cats appears to be low.

b. No evidence-based recommendations can be made regarding the use of antithrombotic therapy in cats with arterial catheters.

c. We suggest antithrombotic therapy should not be routinely used in cats with arterial catheters.

1.20. Vascular access ports (Dogs) [16/16 Round 2]

a. There is insufficient evidence to determine whether the use of vascular access ports in dogs increases risk of thrombosis.

b. No evidence-based recommendations can be made regarding use of antithrombotic therapy in dogs with vascular access ports.

1.21 Vascular access ports (Cats) [19/19 Round 1]

a. There is no evidence that use of vascular access ports in cats is associated with an increased risk of thrombosis.

b. We suggest antithrombotic therapy should not be routinely used in cats with vascular access ports.

1.22. Extracorporeal circuits (Dogs) [16/16 Round 2]

a. Extracorporeal circuits are associated with activation of coagulation and circuit thrombosis in dogs, necessitating use of systemic or regional anticoagulation during extracorporeal procedures unless otherwise contraindicated.

b. The risk of systemic thrombosis in dogs between extracorporeal therapy cycles appears low.

c. We suggest that antithrombotic therapy should not be routinely used between extracorporeal therapy cycles in dogs unless indicated by other risk factors for thrombosis.

1.23. Extracorporeal circuits (Cats) [16/16 Round 2]

a. Extracorporeal circuits are associated with activation of coagulation and circuit thrombosis in cats, necessitating use of systemic or regional anticoagulation during extracorporeal procedures unless otherwise contraindicated.

b. No evidence-based recommendations can be made regarding use of antithrombotic therapy between extracorporeal therapy cycles in cats.

1.24. Transvenous cardiac pacemaker (Dogs) [14/16 Round 2, 1 abstained, 1 felt the statement strength was too strong]

a. Transvenous cardiac pacemaker implantation is weakly associated with symptomatic thrombosis in dogs, while lead-associated thrombosis is more common.

b. We recommend use of antithrombotic therapy in dogs following transvenous pacemaker implantation where other risk factors for thrombosis exist.

c. We suggest that antithrombotic therapy can be considered in all dogs following transvenous pacemaker implantation.

1.25. Transvenous cardiac pacemaker (Cats) [15/16 Round 2, 1 abstained]

a. No evidence-based recommendations can be made regarding the use of antithrombotic therapy in cats following transvenous cardiac pacemaker placement.
